# Genetic Foundation of Leaf Senescence: Insights from Natural and Cultivated Plant Diversity

**DOI:** 10.3390/plants13233405

**Published:** 2024-12-04

**Authors:** Phan Phuong Thao Doan, Hue Huong Vuong, Jeongsik Kim

**Affiliations:** 1 Interdisciplinary Graduate Program in Advanced Convergence Technology & Science, Jeju National University, Jeju 63243, Republic of Korea; 2 Subtropical Horticulture Research Institute, Jeju National University, Jeju 63243, Republic of Korea; 3 Faculty of Science Education, Jeju National University, Jeju 63243, Republic of Korea

**Keywords:** senescence, genetic diversity, association analysis, accessions, cultivars, GWAS, omics

## Abstract

Leaf senescence, the final stage of leaf development, is crucial for plant fitness as it enhances nutrient reutilization, supporting reproductive success and overall plant adaptation. Understanding its molecular and genetic regulation is essential to improve crop resilience and productivity, particularly in the face of global climate change. This review explores the significant contributions of natural genetic diversity to our understanding of leaf senescence, focusing on insights from model plants and major crops. We discuss the physiological and adaptive significance of senescence in plant development, environmental adaptation, and agricultural productivity. The review emphasizes the importance of natural genetic variation, including studies on natural accessions, landraces, cultivars, and artificial recombinant lines to unravel the genetic basis of senescence. Various approaches, from quantitative trait loci mapping to genome-wide association analysis and *in planta* functional analysis, have advanced our knowledge of senescence regulation. Current studies focusing on key regulatory genes and pathways underlying natural senescence, identified from natural or recombinant accession and cultivar populations, are highlighted. We also address the adaptive implications of abiotic and biotic stress factors triggering senescence and the genetic mechanisms underlying these responses. Finally, we discuss the challenges in translating these genetic insights into crop improvement. We propose future research directions, such as expanding studies on under-researched crops, investigating multiple stress combinations, and utilizing advanced technologies, including multiomics and gene editing, to harness natural genetic diversity for crop resilience.

## 1. Introduction

Leaves are the plant’s main photosynthetic organs and are critical for its growth. Leaf senescence occurs at the final stage of leaf development in a genetically well-controlled manner and is considered a model for the study of plant senescence [1]. This destructive process involves nutrient remobilization from old leaves to newly developed organs or storage tissues to ensure the maintenance of plant fitness and reproductive success. During senescence, chlorophyll (Chl) degradation leads to visible leaf yellowing, signaling the breakdown of the photosynthetic machinery and the redistribution of essential macronutrients, such as nitrogen (N), phosphorus (P), and potassium (K) [2,3]. During senescence, carotenoids undergo transformation, characterized by the accumulation of secondary carotenoids and a decline in primary photosynthetic carotenoids. This transformation contributes to the vibrant color changes associated with leaf senescence. The biological significance of leaf senescence extends beyond nutrient recycling for the next generation; it is a key adaptive strategy that enables plants to optimize resource allocation in response to environmental pressures, such as abiotic (e.g., water and nutrient deficiency, extreme temperature, and day-length) and biotic (e.g., pathogen infection) stresses. Thus, leaf senescence is a highly regulated process that maximizes plant survival under changing environments and contributes to crop production.

Leaf senescence involves highly complex genetic programs that are tightly tuned by multiple regulation layers. Significant advances have been made in the past decades to elucidate the multilevel regulation of leaf senescence, including transcriptional, post-transcriptional, translational, and post-translational mechanisms [1,4,5]. Numerous transcription factors (TFs), among which NAC and WRKY are extensively studied, have been identified as involved in leaf senescence regulation [6]. Furthermore, accumulating evidence has demonstrated that leaf senescence is also under epigenetic regulation, including DNA methylation, covalent histone modifications, and chromatin remodeling [7]. Therefore, understanding the molecular and genetic regulation of leaf senescence is critical for basic plant biology and the manipulation of this trait in agronomically important plants [8].

Genetic analyses of the natural variation underlying senescence in plants are mainly performed by quantitative trait loci (QTL) linkage mapping, where phenotypic variation is associated with the allelic variation in molecular markers segregating in experimental mapping populations derived from directed crosses [9]. The first conventional molecular markers used to link traits to genetic loci in QTL mapping were restriction fragment length polymorphisms (RFLPs), simple sequence repeats (SSRs), and amplified fragment length polymorphisms (AFLPs). Developing higher-resolution molecular markers, particularly single nucleotide polymorphisms (SNPs), enabled by next-generation sequencing (NGS), exome sequencing, and restriction site-associated DNA sequencing (RAD-seq), further advanced the field. These cutting-edge technologies have been applied to a broader range of populations, including biparental populations, multiparent advanced generation intercross (MAGIC) populations, and extensive collections of natural accessions or commercial varieties. This has led to more robust association analyses of genetic loci in standardized populations, particularly through genome-wide association studies (GWAS). The recent advancements in gene-editing technologies, such as clustered regularly interspaced short palindromic repeats associated with CRISPR-associated protein 9 (CRISPR/Cas9), have significantly contributed to this field, providing new opportunities to evaluate the effects of genes across diverse genetic backgrounds.

This review aims to highlight the significant contributions of natural diversity analysis to our understanding of leaf senescence, emphasizing the insights gained from *Arabidopsis thaliana* and major crop species. We will summarize how studies leveraging natural variation have uncovered novel genetic regulators and pathways involved in senescence and how these findings can improve plant fitness and agronomic traits in crops. Additionally, the review examines the existing challenges in applying these genetic insights to practical crop improvement strategies. We suggest future research directions, such as expanding studies to include a diverse array of ecologically and agronomically important species, combining multiple stress factors, and advancing technologies, such as multiomics and gene editing. Our goal is to better understand the implications of senescence and improve crop resilience and yield through the utilization of genetic diversity.

## 2. Role of Leaf Senescence in Plant Adaptation and Crop Yield Optimization

Leaf senescence is a highly regulated developmental process that is crucial for nutrient remobilization from senescent leaves to support the growth and development of younger leaves, flowers, and seeds [10]. Although senescence is inherently a deteriorative process, it is central to plant growth, reproductive success, and overall fitness. Leaf senescence is a tightly coordinated process regulated in a timely manner by transcriptional control. The onset of senescence involves the upregulation of senescence-associated genes and the downregulation of photosynthetic genes, leading to visible symptoms like Chl degradation and leaf yellowing. Chl degradation is mediated by the “PHEOPHORBIDE *A* OXYGENASE (PAO)/phyllobilin” pathway, a highly conserved enzymatic process that converts Chl into nontoxic catabolites [11]. Key enzymes, such as PHEOPHYTINASE (PPH) and PAO, are crucial in this process, ensuring that Chl breakdown does not result in the accumulation of reactive oxygen species (ROS), which can cause cellular damage [12]. Additionally, the autophagy pathway, which involves the degradation and recycling of cellular components within the vacuole, is vital in nutrient recycling during senescence [13]. This metabolic reprograming includes the decline in primary metabolites like sugars and amino acids, while secondary metabolites, such as anthocyanins and flavonoids, accumulate, potentially protecting cells from oxidative damage [14].

Leaf senescence is mainly controlled by developmental age, while other internal and external factors also influence its onset and progress [1,15,16]. Plant hormones are major regulators at each senescence stage, including the initiation, progression, and termination. Hormones like ethylene, jasmonic acid, salicylic acid (SA), abscisic acid (ABA), and strigolactones (SLs) act as senescence promoters, while cytokinins (CKs), gibberellic acid, and auxin are inhibitors that delay the process [17]. The initiation of reproductive development further accelerates senescence by increasing the plant’s sink demand, redirecting resources from the leaves to the reproductive organs [18]. Environmental factors also play a pivotal role in regulating the onset of senescence. Seasonal cues, including changes in day length or temperatures, are critical triggers, with shorter days and cooler temperatures often signaling the need for senescence [19]. Additionally, external expressors, such as drought, salt, DNA damage, extreme temperatures, darkness, and nutrient deficiency, and biotic stresses, such as pathogen infection and phloem-feeding insects, can induce or accelerate senescence [16,20,21]. These abiotic and biotic factors can disrupt cellular homeostasis, leading to the premature degradation of leaf tissues in an effort to reallocate resources to the plant’s more critical parts.

From an evolutionary perspective, leaf senescence is a crucial adaptive mechanism that enhances plant fitness by reallocating resources under variable environmental conditions [4]. In annual plants, the senescence timing is critical to optimize reproductive success; nutrients are redirected from senescing leaves to seeds, enhancing seed viability and vigor and ensuring plant survival over a harsh period [22]. For perennial plants, seasonal senescence allows leaf shedding under unfavorable conditions, such as winter or drought, thus, conserving energy and water [23]. Senescence is also tightly linked to the plant’s ability to adapt to the surrounding environmental conditions in habitats. For example, drought, high temperature, nutrient deficiency, UV radiation, and pathogen or pest infections can induce or accelerate senescence, triggering the premature degradation of cellular components to support the plant’s survival. The adaptive value of senescence is highly context-dependent [24]. Early senescence can confer an advantage in harsh conditions by shortening the plant’s life cycle, directing accumulated energy toward producing healthier seeds that preserve the species’ survival as resilient seeds. In contrast, delayed senescence, or the “stay-green” (SG) trait, prolongs photosynthetic activity, enhancing biomass production and ultimately leading to greater seed yield under more favorable conditions.

The timing and progression of leaf senescence are critical determinants of crop yield, quality, and postharvest stability in agricultural systems. Premature senescence, often triggered by biotic or abiotic stresses, reduces the leaves’ photosynthetic capacity, limiting the plant’s ability to produce and store carbohydrates. This can severely impact yield, particularly in grain crops like wheat (*Triticum aestivum*), rice (*Oryza sativa*), and maize (*Zea mays*), where early senescence leads to reduced grain filling and lower grain weight [25]. Conversely, the SG phenotype, which delays senescence, is central to maintaining photosynthetic activity during crucial stages like grain filling. This trait has been successfully harnessed in breeding programs to improve yield stability, especially under stress-prone conditions [26,27,28]. Identified in crops, including sorghum (*Sorghum bicolor*) and maize, the SG varieties prolong Chl retention, extend photosynthetic activity, and increase biomass accumulation. In leafy crops, where leaves are the main target biomass, this trait also contributes to postharvest longevity by preserving freshness and marketability [29]. The molecular basis of the SG trait has been linked to the regulation of Chl degradation, chloroplast stability, and the maintenance of photosynthetic gene expression during senescence, with key genes like *STAY-GREEN* (*SGR*) and *NON-YELLOW COLORING 1* (*NYC1*). These genes have become targets for genetic manipulation to optimize senescence timing and improve yield and postharvest quality.

However, senescence presents a trade-off between yield and nutrient remobilization. While delaying senescence may maintain leaf photosynthesis capacity to increase yield, it may decrease the efficiency of nutrient remobilization, thus, decreasing produce quality. Conversely, early leaf senescence may render more nutrients in the leaves to be remobilized, increasing the product’s nutritional value, but it may shorten the active duration of leaf photosynthesis, decreasing yield [30]. This trade-off is particularly evident in staple crops, such as wheat, barley (*Hordeum vulgare*), and soybean (*Glycine max*), where a negative correlation has long been observed between grain yield and quality (i.e., protein content) [31,32]. Achieving an optimal balance between these conflicting outcomes requires precise manipulation of senescence timing to maximize photosynthesis without compromising nutrient allocation. Therefore, understanding the molecular mechanisms of senescence regulation is essential to improve grain productivity and quality by balancing the senescence process with a suitable grain filling duration in a certain environment [30].

## 3. Foundational Principles and Current Technologies for Investigating Leaf Senescence Diversity in Plant Populations

### 3.1. Sources of Genetic Diversity

Genetic variation in plants can be broadly categorized into four main sources: natural accessions, landraces, modern cultivars, and artificial recombinant lines. Natural accessions encompass wild populations that have evolved through natural selection and exhibit various morphological traits, such as flower shape and root architecture, and physiological features, such as stress tolerance, flowering period, and leaf senescence [24,33,34]. These populations offer key insights into the adaptive mechanisms that shape ecological and evolutionary dynamics, providing a foundational understanding of the genetic basis for plant adaptation across diverse environments.

Landraces are traditional varieties that have been cultivated and maintained by farmers over generations and represent an essential source of genetic diversity. They are often highly adapted to local environmental conditions and agricultural practices, exhibiting a range of resilience to abiotic stresses, including drought and salinity, and biotic stresses, such as pests and diseases [35,36,37]. Landraces represent a reservoir of genetic diversity that contributes to sustainable agriculture and offers a rich resource for breeding programs aimed at improving stress resilience.

Modern cultivars have been developed through systematic breeding efforts to meet specific agricultural demands, such as higher yield, better grain quality, and increased resilience. They often incorporate beneficial alleles from natural accessions and landraces [22,38]. One notable example is the SG trait, characterized by delayed leaf senescence and prolonged photosynthetic activity. This trait, found in major crops like wheat, maize, rice, sorghum, and barley, significantly contributes to biomass accumulation and grain yield [27]. Since crop domestication, genetic variation among species has been harnessed to improve agricultural adaptation through breeding and genetic manipulation. While domestication and breeding have continuously harnessed genetic variation, modern cultivars focus on optimizing agricultural adaptation under contemporary environmental conditions.

Artificial recombinant lines, such as recombinant inbred lines (RILs) and MAGIC populations, are invaluable tools for studying genetic diversity generated through controlled breeding strategies. RILs, produced by crossing two parental lines and successive selfing or sibling mating, capture recombination events that enable the mapping of QTLs for traits, including leaf senescence [39,40]. The genetic variation in RILs is crucial for dissecting complex traits and understanding the genetic architecture underlying them. However, a significant limitation of the biparental QTL approaches is their restricted genetic diversity and low resolution. In contrast, MAGIC populations, resulting from the intercrossing of multiple parental lines in structured breeding schemes, significantly enhanced genetic diversity and recombination. These populations are essential for the high-resolution mapping of polygenic traits, capturing rare alleles, and exploring complex genetic interactions related to leaf senescence [41,42,43].

Today, many resource centers worldwide curate extensive germplasm collections, preserving the genetic heritage of various plant species. Such centers include the Arabidopsis Biological Resource Center for Arabidopsis, the Rice Genetic Resources Stock Center and AfricaRice GenBank for rice, the Wheat Genetics Resource Center for wheat, the C.M. Rick Tomato Genetic Resources Center for tomato (*Solanum lycopersicum*), and the Maize Genetics Cooperation Stock Center for maize [44,45,46,47,48,49]. They house a wealth of genetic and phenotypic diversity, essential to advance genetic research and breeding efforts.

### 3.2. Genetic and Molecular Markers

Deciphering the genetic basis of natural variation is essential to understanding plant evolution and enhancing resilience and productivity in agricultural contexts. Early genetic studies employed traditional genetic markers, such as RFLPs, AFLPs, and SSRs, which relied on PCR and restriction enzymes, to identify genetic differences, laying the foundation for genetic mapping efforts [50,51,52]. Although these markers span large chromosomal regions, typically ranging from several hundred kilobase pairs (kbp) to megabase pairs (Mbp), they are highly useful in large, diverse germplasm populations. They were instrumental in early QTL mapping efforts, where genetic markers were linked to specific traits across broad genomic regions. QTL mapping, frequently performed in RIL or near-isogenic line (NIL) populations derived from parental lines with distinct phenotypic traits, provided key insights into the genetic architecture of complex traits, including leaf senescence [39,40]. While QTL analysis bridges the gap between genotype and phenotypes, identifying the specific genes underlying these QTLs has been challenging, with only a few cases of successful molecular identification.

The emergence of higher-resolution molecular markers, particularly SNPs, has revolutionized genetic analysis. NGS technologies enable the acquisition of genome-wide sequence information from diverse plant populations, facilitating the generation of extensive SNP datasets across entire genomes. Additionally, targeted sequencing approaches, such as exome sequencing and RAD-seq, provide a cost-effective and efficient way to genotype specific populations, making high-resolution genetic studies accessible even to individual labs. These genome-wide technologies have significantly advanced the field by enabling more detailed and accurate genetic analysis [53,54,55,56]. Moreover, genome-wide sequence information allows researchers to track sequence-level changes in specific genes, gene families, or the entire genome during adaptive evolution, facilitating comparative genetic analysis to better understand genetic and functional diversity. These fine-scale resolutions are also invaluable in molecular breeding, where these markers can be used to introduce desirable traits into crop varieties, such as stress tolerance or prolonged leaf senescence. These advanced approaches have opened up new avenues for studying complex traits in plant populations by providing a finer resolution and more accurate mapping of genetic variation. They are particularly valuable in elucidating the genetic basis of polygenic traits, such as leaf senescence, by capturing common and rare alleles influencing such traits.

### 3.3. Association Analysis

The advent of NGS technologies has also enabled the use of large-scale association studies, such as GWAS and metabolite-based GWAS (mGWAS), to identify genetic loci by analyzing the relationship between genomic variation and traits across diverse populations [57,58]. GWAS have been widely applied in plant research to uncover the genetic basis of complex traits by correlating genetic markers with trait variation. This approach enables high-resolution mapping of loci associated with desirable traits, facilitating the identification of candidate genes for further functional studies. For instance, GWAS have successfully identified genes involved in leaf senescence in several species, such as Arabidopsis, rice, maize, sorghum, and cotton (*Gossypium hirsutum*) [24,59,60,61,62].

Leaf senescence involves a series of coordinated processes with dynamic shifts in metabolites, including changes in anthocyanin and Chl, the distribution of nutrient levels, and changes in senescence-related phytohormones. These processes have been explored through metabolomic approaches [63,64]. mGWAS extends traditional GWAS by linking genomic variation to metabolite profiles, offering novel insights into the genetic regulation of metabolic shifts during senescence [65]. Furthermore, phenomics, employing advanced imaging and sensor technologies, enables the time-series monitoring of a broad spectrum of physiological and metabolic changes throughout senescence [66,67]. By integrating senescence-related traits with principal component analysis or focusing on individual traits from different pathways, such as Chl loss and anthocyanin accumulation, researchers can more comprehensively understand senescence. This approach can replace traditional visual markers in association studies, such as leaf color, providing a more nuanced evaluation of senescence [67,68].

Large population collections enable association analyses that go beyond SNP correlations, linking environmental factors and physiological responses to senescence. Senescence is a tightly regulated degenerative process influenced by internal and external cues [16]. Its role in optimizing plant fitness suggests that the divergence of senescence programs reflects evolutionary adaptations to diverse environments. Additionally, as the final stage of plant development, senescence integrates life-long developmental and physiological responses, evolving through natural selection [69]. Association analyses provide a powerful framework to investigate the environmental and physiological factors shaping senescence programs [70]. By examining genotype–environment interactions, researchers can uncover the selective pressures and adaptations driving the evolution of senescence across populations [66,67]. Furthermore, haplotype-based association studies of key senescence regulators offer insights into these genes’ functional roles, revealing how specific regulatory elements contribute to senescence variation within accessions that carry those haplotypes. This approach enhances our understanding of the complex genetic architecture underpinning senescence and its adaptive significance.

### 3.4. Functional Analysis of Genetic Loci

Functional analysis of the genetic loci underlying leaf senescence is key to understanding how allelic variation in natural accessions or cultivars contributes to this complex trait [71]. By examining allele-specific effects, researchers can gain insights into the adaptive or selected function of alleles on loci that regulate senescence onset, progression, and physiological responses. This approach enhances our understanding of how natural genetic diversity influences senescence timing for stress adaptation and crop improvement [67].

Overexpression studies are valuable for evaluating allelic function, but varying expression levels across different accessions make comparisons challenging [72]. Loss-of-function approaches are often more informative. However, conventional knockout techniques, such as T-DNA insertion and targeting induced local lesions in genomes (TILLING), present challenges, particularly in large populations. These methods rely on random mutations and are more suited for standard backgrounds, whereas RNA interference (RNAi) suffers from variable gene silencing across accessions, limiting its broad applicability. These limitations emphasize the need for more robust approaches.

CRISPR/Cas9-based knockout approaches offer a more reliable and versatile alternative to investigate gene functions across various accessions [67,73]. The precision of the CRISPR technology allows for targeted gene disruption, making it easier to generate loss-of-function mutations. This method is especially useful for functional analysis in natural accessions, where genomic variability may hinder conventional approaches. The CRISPR/Cas9 system’s simplicity ensures that multiple accessions can be tested for specific gene roles in leaf senescence with greater ease and consistency.

Therefore, the functional analysis of the genetic loci underlying leaf senescence is vital for understanding natural variations; while conventional methods present challenges, CRISPR/Cas9 technologies offer a promising path forward. These advanced tools enable more precise and efficient gene editing, paving the way for deeper insights into how different alleles regulate senescence across diverse accessions.

**Figure 1:**
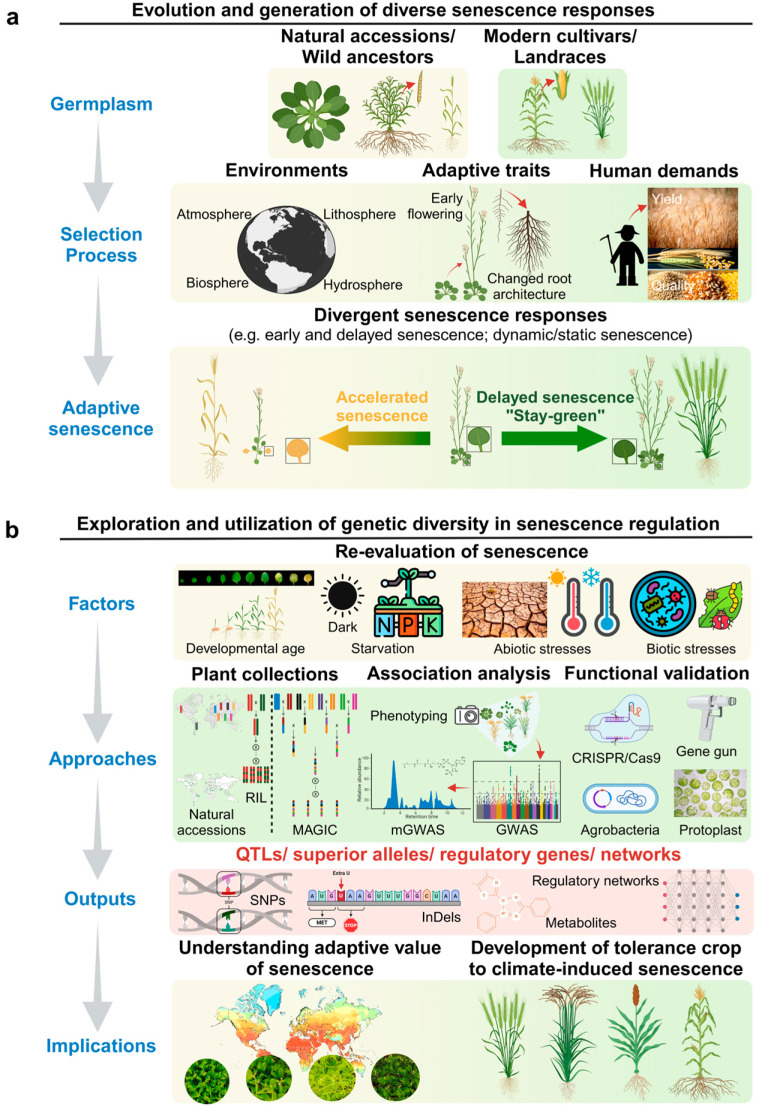
Leveraging Genetic Diversity in Plant Senescence Programs. (**a**) Schematic illustration of senescence diversity in plants. Natural accessions, wild relatives, landraces, and modern cultivars under various environmental conditions (e.g., atmosphere, biosphere, lithosphere, and hydrosphere) evolve distinct senescence programs driven by natural selection or human intervention (e.g., selection for seed yield and quality), often in conjunction with other adaptive traits (e.g., flowering time or root architecture). While natural accessions tend to exhibit early senescence to enhance survival under challenging conditions, domesticated crops have been selected for the SG trait to optimize grain yield and quality. (**b**) Research approaches to uncover the genetic diversity underlying leaf senescence programs. Leaf senescence can be induced by factors like aging, starvation (e.g., low light or nutrient), and environmental stresses, including abiotic (e.g., drought, extreme high or low temperatures) and biotic stressors (e.g., pathogens and pests). Using natural populations, diverse cultivars, and artificial inbred lines (e.g., RILs and MAGIC), researchers can perform genetic association studies and functional analyses to elucidate the adaptive value of senescence traits. This knowledge is crucial to breed crops with enhanced resilience to senescence under unpredictable climates.

In this context, using diverse genetic resources while applying advanced technologies has enabled researchers to pinpoint key genes and regulatory elements that control the timing and progression of leaf senescence (Figure 1). These discoveries deepen our understanding of the biological processes involved and translate into actionable strategies to enhance stress resilience and maintain crop productivity under environmental challenges. The research outputs discussed throughout this review demonstrate how leveraging genetic diversity and innovative technologies can drive significant progress in developing crops with optimized senescence patterns to support global food security under climate change.

## 4. Molecular and Genetic Regulation of Leaf Senescence Using Natural Diversity and Cultivar Variations

Leaf senescence is a crucial phase in a plant’s life cycle, marked by nutrient breakdown and redistribution to support reproductive success [1,10]. Although primarily driven by the plant’s developmental age, senescence is also influenced by environmental conditions and biological factors, such as growth dynamics and flowering transition [66,74,75]. In this context, exploring the evolutionary and adaptive roles of leaf senescence across various accessions and cultivars is essential, as their senescence programs may reflect strategies to enhance fitness in diverse environments [4,71]. Given its significance, the regulation of leaf senescence—especially through the lens of natural genetic diversity—has become a focal point of current research, aiming at uncovering novel mechanisms that plants employ to modulate this process under various environmental conditions.

### 4.1. Age/Natural Development-Induced Leaf Senescence

Studying leaf senescence through natural accessions offers valuable insights into how this trait contributes to plant adaptation and evolution across diverse environments. Arabidopsis has been extensively used as a model organism to investigate the natural variation in leaf senescence, given its wide distribution across various geographic and climatic zones. This diversity reflects an evolutionary history shaped by significant environmental changes.

Many studies have revealed considerable differences in the onset, progression, and intensity of age/natural development-induced leaf senescence among Arabidopsis accessions from different regions, influenced by environmental factors and biological processes associated with these variations [76,77,78,79,80]. Levey and Wingler [77] conducted early investigations to evaluate the relationships between age-induced leaf senescence and various life history traits, including photosynthetic potential, reproductive efficiency, and postbolting longevity, using eight Arabidopsis accessions collected from different continents. They found considerable variations in the timing and duration of leaf senescence and identified day length as a significant factor influencing the process. Their study also linked senescence with photosynthetic capacity and reproductive traits, such as floral transition and seed production, suggesting that senescence mediates the trade-off between maintaining photosynthesis and reproductive success.

Building on these findings, Luquez et al. [78] conducted a more comprehensive study involving 45 Arabidopsis accessions and 155 RILs derived from Cvi × Ler populations. They confirmed that leaf senescence and postbolting longevity are strongly genetically controlled. Their study also revealed an inverse relationship between leaf senescence and flowering time, indicating that Arabidopsis populations may have evolved different strategies for energy allocation depending on their flowering time. Early flowering populations rely on current photosynthates, whereas later flowering populations recycle nutrients from senescing leaves. Additional studies using heterogeneous inbred families and RILs have demonstrated that early-emerging leaves—the first six leaves—and later-emerging leaves have distinct biological roles [76,79,80]. Early leaf senescence primarily contributes to nutrient recycling, including N, to support the growth of later leaves and whole-plant senescence rather than directly aiding reproduction. This observation suggests that senescence induction in Arabidopsis is closely related to floral initiation but not necessarily to later processes, such as silique and seed formation.

Beyond identifying the biological processes related to senescence, biparental mapping populations can reveal the genetic basis of the natural variation in leaf senescence through QTL analyses [68,78,81]. Such studies have isolated several accession- or condition-dependent QTLs associated with senescence traits, suggesting that plants have evolved natural genetic variations shaped by their evolutionary and ecological histories. For instance, QTL mapping in the 415 RILs of the Bay-0 × Sha population was used to explore the genetic basis of Chl degradation and anthocyanin accumulation during leaf senescence [68]. Notably, the absence of overlap between QTLs associated with leaf yellowing and those linked to anthocyanin-induced redness suggests that these two processes are governed by genetically independent pathways. This observation implies that these senescence-related coloring traits may have evolved independently, potentially under different environmental conditions and at different times.

More recently, three Arabidopsis RILs between Col-0 and Ct-1, Cvi-0, or Bur-0—to avoid the limited genetic diversity present in biparental RILs—were used to map QTLs associated with ten traits related to senescence, resource allocation, and reproduction [81]. Subsequent QTL metanalyses identified key regions of metaQTLs where the QTLs for several traits colocalized. These regions were further compared with the positions of candidate genes involved in senescence and flowering time, offering a comprehensive genetic model comprising 13 metaQTLs corresponding to known regulatory genes. Despite this progress, significant variations remain across different biparental RIL populations regarding the relationships between leaf senescence, N use efficiency, rosette biomass, yield, and flowering-related traits. This information suggests that the genetic control of leaf senescence has evolved in a highly complex and population-specific manner.

QTL analyses using RILs derived from two parents exhibiting distinct senescence phenotypes have been widely conducted across various crops, including rice [82,83], wheat [84,85], maize [86,87], sorghum [88,89], and asparagus bean (*Vigna unguiculata*) [90], to identify the genetic loci and molecular markers associated with age-related SG traits. In rice, F2 and RIL derived from the functional SG japonica cultivar (cv.) SNU-SG1 and the high-yielding Tongil-type cv. Milyang23 were employed to identify three major QTLs associated with two SG traits: the degree of mean Chl content in the flag and second leaves (DCFS) and the degree of Chl content in the flag leaf at the heading date (DCF) [82]. These QTLs were mapped to two chromosomes (Chr), with *DCFS7* located on Chr 7 and *DCFS9* and *DCF9* on Chr 9, potentially controlling the maintenance of the flag leaf’s greenness. Furthermore, an extended study utilized two independent RIL populations from the intra- and intersubspecific crosses, specifically between two japonica cv., SNU-SG1 and Suweon490, and between SNU-SG1 and the indica cv. Andabyeo [83]. QTL mapping based on SSR markers identified six QTLs associated with two SG traits related to the Chl content of the flag leaf (CSFL) and the four upper leaves (TCS) across both populations. Among these, four QTLs, including *TCS4*, *CSFL6*, *CSFL9*/*TCS9*, and *CSFL12*, were consistently mapped to Chr 4, 6, 9, and 12, respectively. In wheat, three QTLs associated with SG were identified on Chr 1AS, 3BS, and 7DS, using a RIL population of SG cv. Chirya 3 and non-SG cv. Sonalika [84]. In temperate elite maize, F2 mapping populations generated by crossing an SG line PHG39 (in private breeding) with two non-SG lines, B73 (Corn Belt Dent) and EA1070 (European flint), were used for QTL detection [87]. This study identified several molecular markers associated with the SG trait on Chr 1, with *BNLG1556* contributing the most to the discrimination between early and late senescing genotypes. In asparagus bean, a population of 209 RILs derived from a cross between SG variety ZN016 and non-SG variety ZJ282 was used for QTL mapping of four horticultural traits such as days to first flowering, nodes to first flower, leaf senescence, and pod number per plant [90]. For leaf senescence, a major QTL, *Qls.zaas-11*, was identified, explaining 29% of the phenotypic variation. Notably, the ZN016 allele at *Qls.zaas-11* conferred delayed senescence and a higher pod number per plant, suggesting its potential utility for introgression in breeding programs. Overall, these studies provided valuable QTL target regions for the marker-assisted selection of the functional SG trait, presenting a promising strategy to improve crop yield.

Recent advances in understanding the genetic basis of age-induced senescence have further illuminated the genetic mechanisms underlying SG traits. For example, the rice cv. CR2002, developed from an interspecific cross between *O. sativa* cv. Hwaseong and the wild species *O. grandiglumis*, exhibited an SG phenotype and high grain weight [91]. This phenotype is attributed to the potential low activity of the *O. grandiglumis* allele at the *qCC2* locus. Through fine mapping and subsequent functional validation using knockout lines, *qCC2* was identified as *GRAIN WIDTH AND WEIGHT 2* (*OsGW2*), which encodes a RING-type E3 ubiquitin ligase and is a QTL controlling grain width and weight [92]. This discovery underscores the role of *OsGW2* as a post-translational regulator that modulates delayed senescence and increased grain yield—traits that have been selectively favored during domestication. In another study, Shin et al. [93] identified natural alleles that regulate delayed senescence in japonica and early senescence in indica cultivars. QTL analysis and subsequent map-based cloning revealed a promoter variation in indica that causes an increased and earlier induction of the *OsSGR* gene, resulting in accelerated senescence and shorter lifespans. The indica-type promoter is found in the progenitor subspecies *O. nivara*, indicating its early acquisition during the evolution of the rapid cycling trait in the rice subspecies. Japonica *OsSGR* alleles introgressed into indica-type Korean cultivars delayed senescence, with increased grain yield and enhanced photosynthetic competence. This finding highlights the importance of the *OsSGR* promoter diversity in breeding high-yield rice varieties.

In wheat, two segregating lines—178A and 178B—derived from one of the Chinese wheat cv. Xiaoyan 54 and Jing 411 RIL populations exhibited distinct differences in leaf senescence dynamics and yield-related traits under field conditions [85]. The QTL analysis of a subsequent RIL set from 178A and 178B identified three major QTLs associated with green leaf duration during the grain-filling stage. Fine mapping of the *qGLD-6A* locus identified *NO APICAL MERISTEM-A1* (*TaNAM-A1*) as the causal gene. Functional validation using transgenic lines and TILLING mutants revealed that *TaNAM-A1* promotes leaf senescence by upregulating genes involved in macromolecule degradation and nutrient remobilization, including iron and zinc. In addition to its role in senescence, *TaNAM-A1* affects spike length and grain size. The study demonstrates that natural variations in *TaNAM-A1* result in functional divergences in its ability to transactivate downstream genes, leading to differences in the leaf senescence timing among wheat cultivars.

However, the main limitations of biparental QTL approaches include their low genetic diversity and poor genetic resolution. Consequently, the recent development of MAGIC populations—generated by intercrossing multiple founder lines over several generations—represents a significant advancement in the study of natural genetic variations [41,94]. Although MAGIC populations were first developed in Arabidopsis, their application to the study of leaf senescence remains underexplored. The utility of MAGIC populations in studying leaf senescence has been first demonstrated in bread wheat [42]. ‘NIAB Elite eight-founder MAGIC’ populations, comprising Alchemy, Brompton, Claire, Hereward, Rialto, Robigus, Soissons, and Xi-19, were used to evaluate the genetic elements that influence the timing of the developmental stages in the European elite winter varieties, based on approximately 18,000 SNPs. Notably, researchers identified the genetic components and physiological traits associated with variations in the timing of the key phenological stages of senescence. For example, the timing of senescence was highly correlated with the interval between GS55 (ear 50% emerged) and FLS (onset of flag leaf senescence), indicating a developmental linkage between the period from flowering to senescence and the onset of senescence. Additionally, a negative correlation was observed between FLS and the first flag leaf’s length, indicating a compensatory role between photosynthetic activity in flag leaves and catabolic processes in senescing leaves. FLS was also negatively correlated with disease resistance, implying a potential involvement of biotic stress responses in the senescence response. Further QTL mapping of the FLS trait revealed that the most prominent QTL was found on Chr 5A. Additionally, the same group later identified other QTLs for senescence on Chr 2D, 4D, and 7B, although their molecular identities remain to be determined [95].

Another study was performed on maize using 672 MAGIC populations derived from eight founders, with around one million SNPs, by examining senescence-related traits, such as Chl contents and Fv/Fm (maximum quantum yield of photosystem II), and agronomic traits, including grain yield and flowering time [43]. The study identified 36 candidate genes through GWAS, with 11 involved in key senescence-related processes, such as proteolysis, sugar transport, and sink activity. Notably, *Zm00001d043586*, homologous to the *AtS40-1* senescence regulator, was strongly linked to Chl degradation two months after silking, underscoring its role in late-stage senescence. This study emphasized the genetic complexity of natural senescence in maize.

In potato (*Solanum tuberosum*), the MASPOT population—a MAGIC population derived from a poly-parental cross of 18 elite tetraploid cultivars—was genotyped via sequencing, yielding 93,170 SNPs. This panel of 762 offspring were subsequently used for GWAS on five key traits, including an earliness proxy for senescence, yield, dry matter content, length-to-width ratio, and chipping quality [96]. For the senescence-related trait, two significant SNP regions were identified: one on Chr 2, mapping to the *PGSC0003DMG400012642* gene, which explained 6% phenotypic variance, and a major peak spanning 10 to 60 Mb on Chr 5, showing with a strong association and explaining 14% phenotype variance. Notably, the genomic regions on Chr 5 include the *CYCLING DOF FACTOR 1* (*StCDF1*), a member of the plant-specific DNA-binding with one finger TF family. The natural allelic variants of *StCDF1*, which functions as a mediator between the circadian clock and the tuberization signal, correlated with leaf maturity and senescence onset [97,98]. These findings suggest that *StCDF1* may play a pivotal role in natural variations in senescence in potato.

The establishment of genome-wide SNPs through NGS technology facilitates GWAS as a tool to dissect QTLs underlying complex traits and identify functional genes [57]. Common mapping resources for GWAS include accessions or landrace genetic resources and breeding lines, which have been used to discover the genetic loci responsible for leaf senescence in various model plants and crops [59,62,67,99,100,101]. A pioneering study using GWAS to reveal the genetic basis of natural leaf senescence was conducted in rice. A GWAS conducted with a worldwide collection of 529 *O. sativa* accessions identified 46 significant loci associated with high Chl content [99]. Two pleiotropic genes, *GRAIN NUMBER*, *PLANT HEIGHT*, *AND HEADING 7* (*GHD7*) and *NARROW LEAF 1* (*NAL1*) were confirmed as major loci controlling the natural variation in Chl content at the heading stage through analyzing their NIL and transgenic lines. *GHD7*, a putative HAP3 subunit of the CCAAT-box-binding transcription factor, also functions as a natural genetic variant in traits related to flowering time, growth, and grain yield. This information indicates its essential function in managing and coordinating the dynamic transition between source and sink during rice growth and development, ultimately increasing plant fitness with higher yields [102]. Additionally, the haplotype analysis of *NAL1* revealed diverse effects of SNP variations on flag leaf width and spikelet number, further supporting its role in balancing growth and reproduction for optimal plant fitness [99,103].

Another study utilized a diverse population of 368 rice accessions from 32 countries for a GWAS as part of the 3000 Rice Genome Project, and identified *OsSG1* (LOC_Os07g27790) as a primary natural variant contributing to Chl content and strong SG traits [59]. Further population analyses, including an additional 446 wild accessions, based on SNPs within hundreds of genes associated with Chl content and SG, suggested that several dozen genes underwent strong positive selection in cultivated japonica rice during its spreading from subtropical origin to the North China temperate zone. This finding indicates that during the domestication of japonica, planting areas gradually extended from low to high altitudes, accompanied by changes in light intensity and day length. During this adaptation, new natural mutations for higher Chl content and SG were preserved and gradually accumulated, along with elite variations from wild-type rice.

In maize, senescence phenotypes were evaluated on 392 inbred lines with 438,161 SNP markers from three different environments (Verona, Arlington, and Pendleton in the USA) [100]. Subsequent GWAS identified 64 candidate genes, 14 of which are involved in senescence-related processes, including proteolysis, sugar signaling, sugar transport, and sink activity. Eight of the GWAS candidates, independently supported by a coexpression network underlying SG, include a trehalose-6-phosphate synthase, a NAC transcription factor, and two xylan biosynthetic enzymes. Source–sink communication and cell wall activity as a secondary sink emerge as key SG determinants. A more recent study expanded on these findings by performing a comprehensive analysis of genotypes with contrasting source–sink-regulated senescence (SSRS) phenotypes, focusing on B73 and Mo17 inbred lines with contrasting SSRS phenotypes [101]. This multienvironmental evaluation, using a biparental population and a diversity panel, led to identifying 12 QTLs and 24 candidate genes associated with SSRS. In a case study of sorghum, GWAS applied to a diverse panel of 333 sorghum lines successfully identified haplotypes in 45 key candidate genes associated with senescence phenotypes [62]. The senescence-delaying haplotypes of these genes were under strong selection for genetic improvement during sorghum domestication.

In cotton, a GWAS utilizing 355 upland accessions and 3,015,002 high-density SNPs elucidated the genetic architecture underlying senescence [60]. This study identified a total of 977 candidate genes within 55 senescence-related genomic regions (SGRs), designated SGR1 to SGR55. Gene ontology analysis of these candidate genes indicated significant enrichment in biological processes associated with salt stress, ethylene signaling, and leaf senescence. Within SGR36, which spans approximately 420 kb, the gene *GhMKK9* (*Gohir.A12G270900*), encoding a protein belonging to the MAP kinase kinase family, was identified as a pivotal factor in modulating leaf senescence responses. Functional analysis revealed that the two haplotypes, HAP-1 and HAP-2, resulting from nonsynonymous SNPs, exhibited distinct regulatory effects on senescence. Furthermore, virus-induced gene silencing of *GhMKK9* not only delayed age-induced leaf senescence but also enhanced drought resistance in cotton, offering new insights into the genetic mechanisms of senescence. These findings collectively deepen our understanding of age-induced leaf senescence and offer candidate QTLs or genes for functional genomics and molecular breeding in crops.

In Arabidopsis, multiple independent studies investigating leaf senescence during aging, using biparental QTL analysis or GWAS analysis, have identified a novel locus, *ACCELERATED CELL DEATH 6* (*ACD6*) [67,104]. *ACD6* functions as an age-specific positive regulator of senescence, with a hyperactive allele in Ct-1 relative to Col-0, by modulating the N remobilization efficiency and acts as a common regulatory factor contributing to senescence diversity among natural accessions. *ACD6* encodes a transmembrane protein with intracellular ankyrin repeats, which positively regulates cell death and defense mechanisms, in part through the immune hormone SA, as a nexus for the trade-off between growth and disease resistance in wild populations [67,105,106,107]. Although the SNP variations in ACD6 do not clearly correlate with the geographical distribution pattern among these accessions, the hyperactive *ACD6* allele was found mostly in early accessions, such as Mz-0 and Est-1, suggesting a role for *ACD6* in local adaptation [106,107]. These findings underscore the critical role of *ACD6* in regulating senescence as part of the ecological adaptation mechanisms in natural Arabidopsis accessions. ACD6 links senescence with N remobilization and defense pathways, reflecting the evolutionary trade-offs between growth and survival in wild populations.

These findings deepen our understanding of age-induced leaf senescence in the context of ecological adaptation and domestication. They offer valuable candidate genes for functional genomics and molecular breeding in crops, providing opportunities to optimize senescence regulation for improved agricultural performance. Moreover, studies on the natural diversity of Arabidopsis suggest fundamental strategies for controlling senescence shaped by environmental and biological processes in ecological and evolutionary contexts. These insights enhance our knowledge of plant aging and hold the potential for developing crops with greater resilience and resource-use efficiency.

### 4.2. Starvation-Induced Leaf Senescence

Senescence is a crucial adaptive strategy that reallocates energy and nutrients to support reproductive success, ensuring the survival of future generations. It is highly influenced by environmental factors, such as light availability and nutrient levels, which are central to regulating plant metabolism and development. Particularly, starvation-induced senescence, including light deficiency/dark-induced and nutrient deficiency-induced leaf senescence, provides critical insights into how plants manage resource scarcity [14,108,109].

#### 4.2.1. Light Deficiency/Dark-Induced Leaf Senescence

Light is essential for plant growth, morphology, and development [110]. Consequently, when leaves are exposed to shade or prolonged darkness, leaf senescence is triggered as a life strategy to optimize energy utilization, ensuring reproductive success [111]. Transcriptomic analyses indicated that dark-induced senescence shares many commonly regulated genes with age-induced senescence while also exhibiting dominant changes in sugar catabolism and lipid β-oxidation [112,113]. Additionally, dark treatment is widely employed as a rapid, convenient, and effective method to induce leaf senescence, making it easier to investigate the impact of additional senescence regulators, such as phytohormones, sugars, and secondary metabolites [114,115].

Dark-induced senescence has been investigated using natural populations to uncover the genetic basis of divergent senescence programs. In Arabidopsis, a time-series analysis of quantitative senescence responses under darkness was conducted on 259 natural accessions using a high-throughput Chl fluorescence imaging system [24]. Subsequent GWAS identified nonsynonymous SNPs strongly associated with senescence at a novel locus of *GENETIC VARIANTS IN LEAF SENESCENCE 1* (*GVS1*), which encodes a putative reductase. The two *GVS1* variants, resulting from this nonsynonymous substitution, exhibited functional differences in senescence regulation, suggesting that this SNP represents a critical evolutionary modification influencing the role of *GVS1* in senescence regulation. Moreover, *GVS1* is implicated in oxidative stress sensitivity, with different activities across accessions, highlighting a potential link between leaf senescence and oxidative stress. This study proposes that the functional evolution of *GVS1*, in conjunction with senescence and oxidative stress responses, may be crucial in setting adaptive life history strategies.

In an additional study, a time-resolved mGWAS using a diversity panel of 252 Arabidopsis accessions under darkness characterized the metabolic aspects of dark-induced senescence [65]. The researchers identified six patterns of metabolic shifts and linked them to 215 genetic associations across 81 candidate genes, revealing critical roles for genes involved in the metabolism of glycine, galactinol, threonine, and ornithine. A key finding was the regulation of tyrosine content by TYROSINE AMINOTRANSFERASE 1 (TAT1), which affects both enzyme activity and gene expression in response to darkness. Additionally, specific SNPs associated with THREONINE ALDOLASE 1 (THA1) and *AMINO ACID TRANSPORTER 1B* (*AVT1B*) were demonstrated to be vital for maintaining amino acid levels during senescence. These insights provide a comprehensive model of the metabolic processes underlying dark-induced senescence, highlighting the complex genetic regulation that allows plants to manage internal resources in the absence of photosynthesis, thus, contributing to their survival under darkness stress.

In addition to genetic variation involving changes in the DNA sequence, epigenetic variation also has a significant influence on leaf senescence by altering DNA modifications or small regulatory RNAs, offering an additional layer of regulatory complexity for the plant’s adaptation against environmental challenges [7]. In Arabidopsis, a natural allele, *NATURALLY OCCURRING DNA METHYLATION VARIATION REGION 19* (*NMR19)*, was identified as a key factor in the variation in dark-induced senescence responses among 137 natural accessions [116]. This allele modulates the expression of the *PPH* gene, which is crucial for Chl degradation. In accessions with delayed senescence, the hypermethylation of *NMR19-4* (NMR19-4m) represses *PPH* expression; in early senescence accessions, the hypomethylation of *NMR19-4* (NMR19-4u) is associated with high *PPH* expression levels. Notably, a strong correlation was found between the mean temperature of the driest quarter and the methylation status of *NMR19-4*, suggesting that this epigenetic variation may confer adaptive advantages in hot and dry environments. Accessions with *NMR19-4* hypomethylation may reduce water consumption through decreased photosynthesis or by completing their life cycle earlier, thus, avoiding extreme heat. The findings suggest that *NMR19-4* is a functional natural allele that has evolved to help Arabidopsis adapt to varying climatic conditions, potentially playing a critical role in its survival under climate change.

Darkness, an extreme condition of light deficiency, is a significant factor affecting crop yields in dense canopies and adaptation to seasonal photoperiodic variations. The findings mentioned above reveal the genetic and epigenetic networks, including the key genes *GVS1* and *NMR19-4*, which regulate dark-induced senescence and plant adaptation. Understanding these mechanisms provides valuable targets for breeding programs aimed at enhancing crop resilience and efficiency, offering new strategies to cope with light limitations and climate change.

#### 4.2.2. Nutrient Deficiency-Induced Leaf Senescence

Nutrient starvation induces senescence in older leaves, leading to nutrient remobilization in young or newly developing tissues. Among nutrients, N, P, and K are the essential macronutrients with a crucial role in leaf senescence, and their deficiency suppresses plant growth and accelerates senescence [2]. Deficiencies in these macronutrients can also induce anthocyanin synthesis and a red-purple leaf phenotype, a characteristic partially shared with other nutrient stresses, such as sulfur and magnesium starvation [80,117,118]. For example, Diaz et al. [68] performed a QTL analysis for leaf redness and yellowing under low N conditions using an Arabidopsis Bay-0 × Sha RIL population to investigate the senescence response of plants to low N nutrition. This study proposed that plants respond to N limitation by promoting Chl breakdown and inducing typical leaf senescence symptoms in older leaves to facilitate N remobilization for optimal nutrient allocation or by accumulating anthocyanins, which may protect against light damage and influence leaf lifespan and plant longevity.

Some studies focusing on N use efficiency and deficiency tolerance also revealed the genetic basis of N starvation-induced senescence in diverse Arabidopsis natural accessions [119,120,121]. Sakuraba et al. [119] conducted a GWAS using 52 Arabidopsis accessions grown under N-deficient conditions during the seedling stage; they identified several genomic regions potentially linked to N deficiency-induced leaf yellowing. Notable genes near these SNP peaks included *NITRATE TRANSPORTER 1.1* (*NRT1.1*), *AGAMOUS-LIKE 65* (*AGL65*), ATP-*BINDING CASSETTE G1* (*ABCG1*), and INOSITOL 1,3,4-TRISPHOSPHATE 5/6-KINASE 3 (ITPK3). Polymorphisms in the *NRT1.1* promoter sequence, such as a 7 bp deletion in Gr-5 and an A→C SNP in Ullapool-8, were associated with the *NRT1.1* promoter activation and remained green under N starvation. The *nrt1.1* knockout mutant exhibited accelerated leaf yellowing under N starvation, whereas transgenic plants overexpressing *NRT1.1* retained their greenness. Furthermore, grafted chimeras using *NRT1.1*-overexpressing scions and wild-type rootstocks displayed delayed leaf yellowing under N deficiency, which was absent when the grafting was reversed. These results indicate that *NRT1.1* expression in aboveground tissues is crucial for regulating N starvation-induced leaf yellowing.

In cereal plants, N deficiency can lead to reduced photosynthetic ability in leaf organs, stunted growth, lower biomass production, decreased grain yield, and premature leaf senescence [122,123]. *ABNORMAL CYTOKININ RESPONSE 1* (*ABC1*) encodes a ferredoxin-dependent (Fd)-GOGAT, a key enzyme in the glutamine synthetase/glutamine:2-oxoglutarate amidotransferase cycle, which is essential for N assimilation from soil. This gene exhibits significant differentiation between the japonica and indica rice subspecies [124]. A genetic screen aimed at the short stature and pale green phenotype of the *abc1* mutant, which suffers from defective N assimilation, identified *ABC1 REPRESSOR 1* (*ARE1*), encoding a novel, uncharacterized protein [125]. Further analysis of genetic variation across 2155 rice varieties revealed that the *ARE1* promoter region contained small insertions in a substantial portion of the indica (18%) and aus (48%) accessions, leading to a reduced *ARE1* expression. *ARE1* mutation or low expression enhances N utilization and increases grain yield with delayed senescence under N-limiting conditions. These results suggest that genetic variations in the *ARE1* promoter directly regulate its expression, impacting rice productivity under N stress, which may have been selectively favored during breeding.

Another recent study in rice revealed the genetic basis of N use efficiency and deficiency tolerance using a GWAS combined with transcriptome analysis from 230 rice accessions during the reproductive stage [126]. They identified several key QTLs and novel candidate genes that responded to low N conditions. Among these, *OsNIGT1* (*Os02g22020*), *ZINC FINGER/CCCH* TF (*Os05g45020*), and *MYB* TF (*Os06g43090*) emerged as strong candidates for traits related to N deficiency tolerance and use efficiency. More importantly, *Os06g44900* was predicted to be associated with leaf senescence under N starvation, highlighting its potential role in the plant’s adaptive response to N deficiency. This research provides valuable insights into the molecular mechanisms underlying N starvation-induced leaf senescence. It offers promising targets for breeding rice varieties with improved N use efficiency, which is critical for sustainable agriculture under low-N conditions.

P and K are essential macronutrients critical to plant growth and physiological function, with both being mobilizable from senescing leaves. However, their roles in regulating senescence responses in plant diversity remain less explored compared to N. In a study on P starvation-induced leaf senescence, Yang et al. [127] utilized a population of 201 double haploid lines derived from a cross between Jinmai 47, a low P-resistant wheat cultivar, and Jinmai 84, a high-yield wetland cultivar sensitive to low P. The study identified 157 QTLs associated with Chl content in the flag leaf under different P conditions, explaining 3–32% of the phenotypic variation. Of particular interest, two major QTLs, *Qchl.saw-4D.1* and *Qchl.saw-4D.2*, were identified as novel loci for chlorophyll contents under moderate and low P conditions, accounting for 16% and 20% of phenotypic variation, respectively. For K deficiency-induced leaf senescence, QTL mapping on 20 traits, including Chl content, was performed under both optimal and low K treatments at the seedling stage in wheat [128]. Using 168 doubled haploid lines derived from a cross between the low K-tolerant cv. Huapei 3 and the low K-sensitive cv. Yumai 57, the study identified a total of 65 QTLs distributed across nearly all chromosomes, with each QTL explaining 5% to 40% of the phenotypic variation for each treatment condition. Notably, *QCHL-6D*, associated with chlorophyll concentration, is derived from the low K-tolerant allele from Huapei 3. These findings underscore the relevance of P and K deficiencies in the senescence process and highlight specific QTLs that could be utilized in breeding programs to improve nutrient deficiency resilience in wheat.

Nutrient deficiency—a primary driver of early senescence—has, in particular, led to breeding advancements in nutrient use efficiency (NUE). Selecting high-NUE cultivars not only reduces dependence on nutrient-rich fertilizers but also lowers production costs and minimizes environmental impacts. Moreover, insights into the genetic regulation of nutrient-induced senescence have advanced precision agriculture, enabling targeted nutrient application that aligns with crop developmental stages to optimize yield and resource use.

Starvation-induced senescence in plants, influenced by soil conditions and photosynthetic light, reflects an adaptive response to environmental stresses in habitats or cultivation locations. Through domestication, crops have been selectively bred to optimize nutrient use and light capture, enhancing their ability to manage senescence and improve yield under varied conditions. These adaptations highlight the complex interplay between genetic selection and environmental factors that drive productivity and resilience in agricultural systems.

### 4.3. Stress-Induced Leaf Senescence

Since plants are sessile, their growth and development are particularly sensitive to environmental challenges [129]. Accordingly, senescence and its related processes are time-dependent and responsive to environmental factors, such as water deficit, high and low temperatures, and pathogen infections [130].

#### 4.3.1. Drought Stress-Induced Leaf Senescence

Drought triggers various responses in plants, with leaf senescence being a key survival mechanism. Drought-induced leaf senescence promotes nutrient remobilization from aging and stressed leaves, helping sustain the growth, development, and maintenance of younger tissues, reproductive organs, and developing seeds. Additionally, drought-driven senescence often leads to leaf abscission, which minimizes water loss through transpiration and helps maintain water balance, enhancing the plant’s overall resilience under water-limited conditions.

Drought stress is a major constraining abiotic stress with a significant impact on crop growth and productivity, particularly due to the rapid and drastic changes in the global climate. In this regard, the genetic basis and molecular mechanism of drought stress-induced leaf senescence have been reported in several major crops, including wheat [131,132,133,134,135], barley [136,137,138,139], maize [140,141,142], soybean [143], and sorghum [144].

In wheat, Verma et al. [131] conducted the first mapping of QTLs associated with drought stress-induced flag leaf senescence. The study utilized 48 doubled haploid lines derived from the winter wheat varieties Soissons (France-bred) and Beaver (UK-bred), which exhibited contrasting senescence patterns. Soissons, which displayed delayed senescence and was photoperiod-insensitive compared with Beaver, was identified as a key contributor to drought tolerance. A QTL analysis using AFLP and SSR markers revealed a cluster on Chr 2D associated with senescence-related traits, such as yield and percentage of flag leaf area remaining green (%GFLA) under unirrigated conditions. This Soissons QTL was linked to the extended SG phenotype, a key trait for improving yield under water-limited conditions. Another study explored the molecular QTL markers linked to flag leaf senescence under water stress (WS) in wheat [132]. They identified several QTL molecular markers, including *RAPD* (*Pr9*), *ISSR* (*Pr8*, *AD5*, *AD2*, and *AD3*), and *SSR* (*Xgwm382*), associated with drought tolerance and senescence-related traits. These markers explained 22–73% of the phenotypic variation observed between the biparental populations, drought-sensitive Variant-2 and drought-tolerant Cham-6 genotypes, making them valuable indicators of drought tolerance genes in wheat.

Christopher et al. [134] identified a set of SG QTLs associated with well-watered (WW) and water-limited conditions, using a population of doubled haploid RILs derived from a cross between the SG cv. SeriM82 and the senescent cv. Hartog. The study identified ten major QTLs related to delayed leaf senescence, a key component of the SG phenotype, across diverse environmental conditions. Most QTL regions for the SG traits were constitutively expressed in wet and dry environments, including *QSG.qgw-4A.1* and *4A.2*, *QSG.qgw-4B* (colocated with *REDUCED HEIGHT 1* [*RHT1*]), and *QSG.qgw-4D* (colocated with *RHT2*). However, some QTLs exhibited preferential association with specific environments, such as *QSG.qgw-2A* in wetter conditions and QSG.qgw-5B in dryer conditions, indicating traits with inducible expression in response to water conditions. This research highlights the potential of these QTLs in enhancing wheat resilience to WS and their application in breeding programs aimed at improving yield stability under varying environments.

In barley, drought stress-induced premature leaf senescence and tolerance were investigated among 156 winter barley genotypes at juvenile stages subjected to a four-week drought stress period [145]. GWAS, with approximately 3212 SNPs, identified 70 QTLs associated with drought stress and leaf senescence, with two major QTL clusters located on Chr 5H and 2H. Notably, candidate genes on Chr 5H, such as *ARABIDOPSIS VACUOLAR PYROPHOSPHATASE 1 (AVP1)*, *STRESS-ACTIVATED PROTEIN KINASE 9 (SAPK9)*, and *PROTEIN KINASE RESPONSIVE TO ABA 1 (PKABA1)*, are implicated in senescence or dehydration stresses, underscoring their importance in the genetic regulation of these traits. Further analysis by the same group employed a small-scale expression QTL (eQTL) analysis for 48 genes, including 23 involved in drought stress, 12 linked to senescence, and 11 identified through prior GWAS analysis [138]. A strong correlation between the differential expression of *GLUTAMINE SYNTHETASE 2* (*GSII*) and *pHv_NUCLEAR TRANSCRIPTION FACTOR* Y *SUBUNIT ALPHA* (*pHvNF-Y5α*) during drought-induced senescence and the relative Soil–Plant Analysis Development (SPAD) values, an indicator of Chl content, was observed. A GWAS of the expression profiles for 48 genes identified eight cis-eQTL and seven trans-eQTL, elucidating the genetic variation underlying their transcriptional regulation during drought-induced senescence. The combined findings from these studies suggest that two SNP markers located on Chr 5H, *BOPA1_9766-787* and *SCRI_RS_102075*, hold potential for marker-assisted selection in barley breeding programs aimed at improving drought tolerance [138,145]. More recently, a comprehensive analysis of 34 morphological and physiological traits in 209 spring barley genotypes subjected to drought stress during the vegetative and generative stages provided further insights into drought adaptation [139]. A GWAS on relative trait values, with significant genotype-by-treatment interactions under WW and water deficit conditions, identified over 110 QTLs, explaining more than 50% of the genetic variance observed in these traits. Among these, a QTL on Chr 7H (*SCRI_RS_158234*) was linked to accelerated senescence, suggesting a potential drought escape strategy under stress conditions.

In maize, large-scale phenotyping screened for drought resistance-related traits under WS and WW conditions [140]. The study utilized 550 inbred lines from an association mapping panel comprising tropical, subtropical, and temperate accessions provided by the International Maize and Wheat Improvement Center (CIMMYT) and the Chinese Academy of Agricultural Sciences. The results indicated a positive and significant association between grain yield and plant height, Chl content in the ear, and leaf senescence under WS and WW conditions. Drought-related morphophysiological traits, including SG and grain yield, were evaluated in three tropical biparental populations (CML444 × MALAWI, CML440 × CML504, and CML444 × CML441) from CIMMYT under WW and WS conditions to dissect the genetic basis responsible for these traits. These parental lines, adapted to the tropical and subtropical midaltitude environments in Africa, are considered drought-tolerant. A metaQTL analysis across the three populations identified six constitutive genomic regions, each associated with at least two overlapping traits. Among these, two metaQTL regions on Chr 4.09 and 5.05 were linked to SG and grain yield, highlighting the stress-adaptive nature of the SG trait. These regions were previously associated with leaf senescence [142], maintenance of the leaf green area during postflowering [146], and SG characteristics [86].

In soybean, a GWAS on a collection of 359 soybean accessions from 25 countries across Europe, China, and the USA identified genomic regions and candidate genes involved in responses to short-duration drought stress (SDS) and long-duration drought stress (LDS), with assessments based on four traits: canopy wilting, leaf senescence, maximum absolute growth rate, and maximum plant height [143]. For leaf senescence responses, three SNPs located on three different Chr displayed significant associations under SDS conditions, collectively explaining up to 15% of phenotypic variation. On the other hand, 13 SNPs associated with leaf senescence under LDS conditions were distributed across six Chr explaining 1–14% of phenotypic variation. Several candidate genes were identified near these SNPs, including *CALCIUM-DEPENDENT PROTEIN KINASE 1* (*Glyma.02G192700*), *AtDjA3* (*Glyma.02G193000*) encoding a DNAJ heat shock N-terminal domain-containing protein, *PHOTOSYSTEM I LIGHT HARVESTING COMPLEX GENE 1* (*Glyma.16G145800*), and *HEAT SHOCK TRANSCRIPTION FACTOR A3* (*Glyma.19G191700*), a gene associated with thermotolerance and drought tolerance.

Drought-induced leaf senescence represents a crucial survival mechanism, enabling plants to conserve limited water resources by reallocating nutrients to younger tissues and reproductive organs. Genetic studies in several crops, including wheat, barley, and maize, have revealed key drought adaptation strategies and identified QTLs and molecular markers associated with delayed senescence and drought tolerance. These findings provide valuable insights into the genetic basis of drought adaptation and offer useful tools for breeding drought-resistant crop varieties. Incorporating traits, such as SG, into breeding programs holds great potential for improving crop resilience and yield under water-limited conditions.

#### 4.3.2. Heat/High Temperature Stress-Induced Leaf Senescence

Extremely high temperatures, increasingly frequent due to global climate change, pose a major environmental challenge that induces premature leaf senescence in plants [147]. The onset of heat stress accelerates the breakdown of cellular structures, disrupts photosynthetic efficiency, and leads to ROS accumulation, all contributing to leaf senescence. One of the hallmark processes of heat stress-induced senescence is Chl degradation, which is driven by the destabilization of thylakoid membranes and ROS overproduction in chloroplasts [148]. Ethylene accumulation is another critical component, as heat stress is often linked with increased ethylene biosynthesis, triggering the senescence pathway [149].

Genetic diversity studies aimed at uncovering heat tolerance mechanisms have highlighted several key genes and traits in cereal crops and cool-season perennial grasses. Among cereal crops, wheat is a prominent winter crop particularly vulnerable to heat stress. Heat stress negatively affects wheat production, with global yields estimated to decrease by 6% for every 1 °C rise and even greater loss occurring during the reproductive stage [150]. Therefore, identifying heat-tolerant genes and traits is essential for breeding and developing new wheat cultivars adapted to rising temperatures. Until now, numerous studies have emphasized the role of genetic variations associated with the SG trait in improving wheat yields under heat stress [151,152,153,154,155,156,157].

Vijayalakshmi et al. [153] conducted QTL mapping for eight senescence-related traits under heat stress using 101 RILs derived from a cross between two winter wheat cultivars: the heat-tolerant Ventnor (from Australia) and the heat-susceptible Karl 92 (from the USA). The study identified 16 QTLs associated with heat tolerance and senescence-related traits located on Chr 2A, 6A, and 6B. Notably, the QTLs on Chr 2A, including *Q75%G^h^.ksu-2A* and *Q25%G^h^.ksu-2A* for the SG trait and *QMrs^h^.ksu-2A* and *QTmrs^h^.ksu-2A* for the maximum rate of senescence trait, were linked to the microsatellite marker *Xgwm356* and AFLP marker *XCGT.TGCG-349*. Similarly, QTLs on Chr 6A, such as *Q50%G^h^.ksu-6A* and *QTmrs^h^.ksu-6A*, were associated with the AFLP marker *XCGT.GTG-343*. These QTLs significantly delayed leaf senescence under heat stress conditions and represent promising markers for marker-assisted selection in breeding for heat tolerance. Another study identified 23 QTLs associated with Chl content in 251 RILs derived from heat-tolerant HD2808 and heat-susceptible HUW510 wheat cultivars [154]. These QTLs, located on Chr 2A, 2B, 2D, 5B, and 7A, associated with the Chl content, explained 4%–31% of the phenotypic variation. Particularly, the QTL *Qchc.iiwbr-2A*, linked to the microsatellite marker *GWM372*, was consistently stable under heat stress and nonstress conditions, making it a valuable target for improving heat tolerance in wheat.

In a more recent study, a GWAS on a panel of 199 European elite wheat varieties, using 164,197 high-density SNPs, identified 17 QTLs associated with grain yield and SG traits under post-anthesis heat stress responses [155]. Seven of these QTLs, located on Chr 1B, 1D, 3B, 4A, 4B, 6B, and 7B, were specifically linked to postanthesis heat stress responses through genotype × temperature interaction tests. Among them, the QTLs on Chr 1B, 1D, 4A, and 7B were associated with senescence traits under heat stress, whereas the major QTLs on Chr 3B, 4B, and 6B were associated with kernel weights. Interestingly, the QTL on Chr 4B colocated with the *REDUCED HEIGHT-B1* (*RHT-B1*) gene, previously implicated in drought-induced leaf senescence [134]. This overlap suggests a potential connection between heat, drought stress responses, and dwarfism in regulating leaf senescence.

Cool-season perennial grasses, like crops, are significantly affected by extremely high temperatures in transitional and warm climates, where summer temperatures can often exceed 40 °C. Identifying and evaluating the genotypic variation in thermotolerance within these species is crucial to minimizing heat damage. This approach is essential to breed heat-tolerant varieties and provides valuable insights into the mechanisms of heat stress-induced leaf senescence in perennial plants. Several studies have identified genotypes and genetic variations that can be effectively utilized in breeding programs for these grasses [158,159,160]. For instance, Xu and Huang [158] examined leaf senescence responses to heat stress in two bentgrass species: heat-tolerant rough bentgrass (*Agrostis scabra*) and heat-sensitive creeping bentgrass (*A. stolonifera*). Their findings revealed that *A. scabra* exhibited delayed leaf senescence under heat stress compared to *A. stolonifera*, due to slower ethylene and ABA accumulation and a reduced decline in cytokinins. Another study aimed at identifying QTLs associated with heat stress-induced senescence used a creeping bentgrass biparental RIL population of drought-tolerant L93-10 and drought-sensitive 7418-3 [159,161]. This study identified 32 QTLs across heat tolerance traits, such as turf quality, Chl content, membrane stability, and canopy temperature depression. Several of the QTL regions, located on linkage groups 2.2, 4.1, and 4.2 of the L93-10 and on linkage groups 3.1 and 5.1 of the 7418-3 linkage maps, were associated with multiple traits. These QTLs offer potential markers for marker-assisted selection, helping enhance heat tolerance or improve the understanding of the mechanisms regulating heat-induced leaf senescence in creeping bentgrass.

Consistently, in perennial ryegrass, Zhang et al. [160] conducted a comprehensive study using 98 accessions to investigate the genetic diversity and physiological factors related to heat tolerance and senescence-associated traits. The heat tolerance of these accessions was negatively correlated with electrolyte leakage and positively associated with Chl content and relative water content. These results indicate that heat stress, accompanied by reduced relative water content and compromised cell membrane stability, induces senescence through Chl degradation. Under heat stress, the expression levels of four Chl catabolic genes, including *LpNYC1*, *LpNYC1-LIKE* (*LpNOL*), *LpSGR*, and *LpPPH*, were significantly higher in the heat-sensitive accessions than in the heat-tolerant ones. Furthermore, 8 out of 66 SSR markers were linked to heat stress-induced senescence traits, including Chl content and Fv/Fm. Of these, two Chl-associated markers, *M144* and *rv0941*, were located on Chr 4, while six Fv/Fm-associated markers (*Lp165*, *rv0941*, *DLF008*, *B3C10*, *B3B8*, and *B5E1*) were distributed across Chr 3, 4, 5, and 7. These findings provide a genetic foundation and offer valuable markers for breeding heat-tolerant perennial plant species.

Rising global temperatures seriously threaten plant fitness, particularly by accelerating leaf senescence in crops and perennial grasses. Extensive research has uncovered essential genetic variations and QTLs associated with heat tolerance, highlighting the potential to breed more resilient cultivars. These findings emphasize the importance of leveraging natural genetic diversity to develop heat-tolerant varieties, offering a pathway to mitigating the negative impacts of heat stress on plant growth and productivity while improving overall agricultural sustainability.

#### 4.3.3. Cold/Low Temperature (LT) Stress-Induced Leaf Senescence

Exposure to extreme LTs beyond a plant’s optimal tolerance range represents a significant environmental stress, severely hindering growth and development. Cold stress disrupts key cellular components, including membrane lipids and enzymes, altering membrane fluidity and leading to membrane damage, solute leakage, and impaired metabolic processes. These disruptions, caused by altered enzymatic properties, often result in ROS accumulation, subsequently inducing senescence [162,163]. Cold stress-induced leaf senescence is a tightly regulated process that operates on multiple levels and may contribute to the plants’ acclimation mechanisms [79]. The extent of cold stress effects can be modulated by other environmental factors, such as photoperiod, light intensity, and light quality, which significantly influence the plant’s response [164]. Thus, studies utilizing natural populations or cultivars are vital for a deeper understanding of the mechanisms driving cold stress-induced senescence and the plants’ variability in cold tolerance.

Studies on the natural variation in freezing tolerance have been addressed in Arabidopsis accessions. Early investigations highlighted intraspecific differences in freezing tolerance between geographically distant accessions, Cvi and Ler, revealing significant variations before and after cold acclimation and in response to photoperiodic conditions [165]. A quantitative genetic analysis employing a Ler × Cvi RIL population identified seven *FREEZING TOLERANCE QTL* (*FTQ*). Of these, *FTQ4* exhibited the largest effect under two photoperiod conditions, while five other *FTQ* loci displayed photoperiod-dependent behavior. Notably, *FTQ4* is located near the *C-REPEAT BINDING FACTOR* (*CBF*) gene clusters, including *CBF1*, *CBF2*, and *CBF3*, key TFs involved in regulating cold acclimation. Allelic variation in *CBF2* underlies a major QTL for freezing tolerance, with the low freezing tolerance of the *FTQ4*-Cvi allele linked to a deletion in the Cvi *CBF2* promoter region, resulting in reduced RNA expression of *CBF2*. Further sequencing analysis of *CBF* genes in 50 accessions from the Versailles core collection revealed extensive polymorphisms, some of which correlated with differences in *CBF* and cold-responsive gene expression and, consequently, freezing tolerance [166]. These findings indicate that freezing tolerance in Arabidopsis is governed by a complex genetic network, with allelic diversity at the *CBF* loci playing a partial but significant role in the natural variation observed across accessions.

This natural variation in cold acclimation may also influence leaf senescence regulation. During cold acclimation, comparative metabolome analyses between the freezing-sensitive Cvi-1 and freezing-tolerant Ws-2 accessions revealed the accumulation of various sugars, including glucose, fructose, sucrose, and raffinose, driven by the induction of CBFs and their downstream target genes [167]. Interestingly, cold-acclimated leaves do not exhibit the typical sugar-mediated photosynthesis repression [168]. Furthermore, an increased flux through the sucrose biosynthetic pathway appears to alleviate the photosynthesis inhibition during cold stress [169]. In a study examining senescence in plants of the Bay-0 × Sha RIL, cold acclimation was found to significantly delay leaf senescence, and glucose failed to induce the characteristic senescence response in cold-acclimated plants [79]. These findings suggest a potential interaction between cold acclimation and the metabolic regulation of senescence, highlighting the complex interplay between environmental stress responses and senescence pathways. This regulatory interaction could, for example, increase rosette longevity in biennial and winter annual plants that require vernalization for flowering.

Understanding cold-induced leaf senescence in crops such as rice, maize, and cucumber (*Cucumis sativus*) is crucial because these species are highly sensitive to LT stress, which can significantly impair growth and yield. Cold-induced senescence affects overall crop performance and influences key agronomic traits, including grain quality and fruit development. In rice, Park et al. [170] screened genetic variations for cold stress-induced leaf senescence at the seedling stage using 80 RILs from a cross between Milyang23 (indica, cold-sensitive) and Hapcheonaengmi3 (japonica, cold-tolerant and SG) cultivars. Two QTLs, *qSPA-1* and *qSPA-4*, associated with increased Chl level-related SPAD values were identified from Hapcheonaengmi3, explaining 8% and 16% of the phenotypic variance under LT, respectively. Substitution mapping of the *qSPA-4* QTL within an 810 kb region identified its overlap with *qCTS4*, a QTL that exhibited cold-induced yellowing and stunting from intermittent LT [171]. This overlap suggests that *qSPA-4* may contribute to enhanced cold tolerance in japonica rice.

In maize, a comprehensive analysis was performed to evaluate 406 RILs from a MAGIC population derived from eight inbred founders to identify the QTLs responsible for cold tolerance [172]. The study assessed cold tolerance-related traits under controlled cold treatments or early sowing-induced cold exposure in field conditions, analyzing approximately one million SNP markers at high resolution. Fifty QTLs were significantly associated with the functional senescence-related traits, Fv/Fm, under cold conditions, and these QTLs were concentrated in three genomic regions: bin 2.02–2.04, 7.02, and 10.03–10.06 (http://maizegdb.org/bin_viewer). Among the genes near significant QTLs, *GLOSSY2* in bin 2.02, involved in cold tolerance by forming a protective wax barrier and a desiccation-related protein, and a gene associated with Chl content (*GRMZM2G047065*) in bin 10.03–10.06 are potential candidates contributing to cold-induced senescence tolerance.

More recent studies in cucumber have uncovered genetic variants associated with LT tolerance [173]. In this study, two-week-old seedlings from 173 diverse accessions originating from various geographical regions were exposed to natural suboptimal LT environments. A GWAS targeting yellowing and withering phenotypes identified a nonsynonymous SNP strongly linked to LT tolerance at the *gLTT5.1* locus encoding *CsSGR*. The *CsSGR* knockout mutants generated using the CRISPR/Cas9 system exhibited enhanced LT tolerance, characterized by increased Chl content and ROS accumulation under LT stress. Furthermore, the LT-sensitive haplotype CsSGR^HAP-A^, but not the LT-tolerant haplotype CsSGR^HAP-G^, interacts with CsNYC1 to mediate Chl degradation and accelerate leaf senescence. This LT-sensitive haplotype is predominantly found in high-latitude regions, likely contributing to cucumber cold adaptation in unfavorable environments. Notably, *cssgr* mutants also demonstrated enhanced tolerance to salinity, water deficit, and oomycete *Pseudoperonospora cubensis*. Thus, *CsSGR* represents a promising target gene for breeding cucumber varieties with broad-spectrum stress tolerance, ultimately advancing cucumber molecular breeding efforts.

LT stress poses a major challenge to plant growth by disrupting cellular processes and inducing leaf senescence. Research across species, including Arabidopsis, rice, maize, and cucumber, has revealed the complex genetic responses involved in cold tolerance, including key QTLs and SNPs associated with senescence-related traits. These genetic insights offer valuable resources for breeding programs aimed at enhancing cold tolerance. Leveraging natural genetic variation can improve plant resilience to cold stress, contributing to developing crops better suited to colder and increasingly unpredictable climatic conditions.

#### 4.3.4. Biotic Stress-Induced Leaf Senescence

Leaves are continuously exposed to various biotic stress factors, such as pathogens and pests. Severe or recurrent infections can reduce leaf vitality or trigger cell death, inducing leaf senescence. Plants carefully regulate leaf senescence to maintain overall physiological balance, ensuring optimal conditions for the development of new organs and the survival of offspring. Conversely, the progression of leaf senescence can strongly influence a plant’s susceptibility or resistance to pathogens [174]. Modulating senescence does not affect the nature of the plant–pathogen relationship qualitatively but rather quantitatively [175]. Pathogen resistance in plants is highly sensitive to environmental factors, such as temperature and humidity, implying that biotic stress-induced leaf senescence is often shaped by the plant’s surrounding environmental conditions [176].

Pathogen and pest infections in plants are largely influenced by the host’s physiological state, with different pathogens exhibiting preferences for specific conditions, such as tissue age or metabolic activity. Plant pathogens are typically classified as biotrophs and necrotrophs based on their interaction modes with the host [174,177]. Biotrophic pathogens, such as *Xanthomonas* bacteria and *Blumeria* fugi, derive nutrients from living plant cells with active metabolism and generally favor juvenile plant tissues. In contrast, necrotrophic pathogens, such as *Botrytis* and *Macrophomina* fungi, produce toxins and cell wall-degrading enzymes to destroy plant cells, displaying a preference for senescent tissues. Hemibiotrophic pathogens, such as *Pseudomonas* bacteria and *Phytophthora* oomycete, initially behave as biotrophs during the early infection stages but later adopt a necrotrophic lifestyle as the disease progresses [174,177]. However, insect herbivores, *Trichoplusia* and *Spodoptera*, cause mechanical damage through wounding, while gall-forming insects, such as *Smicronyx* and *Besbicus*, trigger abnormal plant growth responses.

Numerous studies have focused on identifying the genetic diversity related to pathogen resistance, particularly against specific pathogens. For example, research has addressed resistance to biotrophic pathogens, such as powdery mildew by *Blumeria graminis* fungi in wheat [178]. Similarly, necrotrophic pathogen resistance has been studied against pathogens like *Macrophomina phaseolina* fungi in cowpea [179] and *Botrytis cinerea* fungi in tomato [180]. Hemibiotrophic pathogen resistance has been examined in the context of *Pseudomonas syringae* bacteria causing leaf spots in Arabidopsis [181] and *Phytophthora sojae* oomycetes causing stem rot in soybean [182]. Insect herbivore resistance has been revealed against two insect herbivores from distinct feeding guilds including the leaf miner *Tuta absoluta* and the phloem feeder *Bemisia tabaci* in tomato [183]. While some genetic pathways identified in these studies overlap with biotic stress-induced senescence, the detailed molecular responses to specific pathogens fall outside the scope of this review. For a more in-depth exploration of these pathogen-specific interactions, readers should refer to other comprehensive reviews [184,185,186]. Here, we focus on the more generalized biotic stress responses contributing to leaf senescence.

Although comprehensive studies specifically focusing on biotic-induced leaf senescence in natural populations or varieties are limited, senescence-related symptoms, such as the presence or absence of lesions (LES) or the yellowing of older leaves (YEL), have been analyzed as indicators of biotic stress-induced senescence. A pioneering GWAS using LES and YEL in 95 Arabidopsis accessions identified several major alleles contributing to the natural variation in these phenotypes [70]. Notably, *ACD6*, associated with the top SNPs, was linked to LES and YEL, suggesting its involvement in senescence responses related to these traits. It has also been confirmed as a common regulator of age-induced senescence [67]. Other studies on natural variation in the immune system of Arabidopsis identified *ACD6* as a positive regulator of cell death and defense responses, mediating the trade-off between growth and disease resistance in wild populations [105,106,107]. The natural *ACD6*-Est-1 allele confers broad-spectrum resistance to various unrelated pathogens, but this protection often comes with a significant growth penalty, manifested as reduced plant stature and increased cell death or lesion formation in leaves [187]. These findings suggest that *ACD6* is a key regulator of biotic stress-induced senescence, balancing growth and disease resistance.

In crops, necrosis, spotting responses, and yellowing associated with senescence and defense processes have been investigated in several studies such as wheat [188,189,190] and barley [191,192]. For example, in wheat, Li et al. [188] conducted genetic mapping using a cross between the early leaf senescence line M114 and the normal line W301. Bulked segregant analysis combined with RNA-Seq indicated that early leaf senescence in M114 is controlled by a single recessive gene, *EARLY LEAF SENESCENCE 1* (*ELS1*). Interestingly, *ELS1* is the same as the leaf rust resistance genes *LRZH22*/*LR13*/*NECROSIS 2* (*NE2*), which are associated with the fungal pathogen *Puccinia triticina* Eriks [189]. *LRZH22*/*LR13*/*ELS1*/*NE2* encode a nucleotide-binding domain and leucine-rich repeat containing a (NLR) protein that induces programmed cell death and plays a dual role in leaf rust resistance and hybrid necrosis. Among 49 wheat accessions tested, sequence alignment of the NLR alleles revealed two key SNP variations, T1308G and A1684C, distinguishing the leaf rust-resistant allele (TA), such as W301, from susceptible alleles (GC). In another case study, the wheat blast fungus strain Br48 was used to identify a hybrid chlorosis-related locus associated with accelerated senescence and enhanced disease resistance in hybrid chlorosis lines [190]. These lines were derived from interspecific crosses between cultivated tetraploid wheat and the wild diploid progenitor *Aegilops tauschii*. *HYBRID CHLOROSIS1* (*HCH1*) locus was identified on the short arm of Chr 7D as a novel gene inducing hybrid chlorosis in *Triticum* species.

In barley, powdery mildew is an obligate biotrophic fungal disease caused by *Blumeria graminis* f. sp. *hordei*. Investigations into the genetic effects of a second natural variant of the *MILDEW LOCUS O* (*MLO*)-11 allele, designated *MLO-11(cnv2)*, revealed that it exhibits a milder phenotype compared to the standard *MLO-11* allele [191,192]. These studies evaluated senescence-related symptoms and resistance to powdery mildew in the cultivars Westminster (*MLO-11*) and Eth295 [*MLO-11(cnv2)*] in comparison to the powdery mildew-susceptible cultivar Baudin, which carries the wild-type *MLO* allele. Sequence data revealed that *mlo-11(cnv2)* arose through recombination between progenitor *mlo-11* repeat units and the 3′ end of an adjacent *stowaway* miniature inverted-repeat transposable element-containing region. This recombination event resulted in reduced DNA methylation and increased expression relative to *mlo-11*. Physiological analyses showed that *MLO-11* induced necrotic leaf spotting, chlorosis, and premature leaf senescence. In contrast, *MLO-11(cnv2)* conferred complete resistance in adult leaves without spontaneous necrosis or loss of photosynthetic tissue, suggesting a rebalancing selection between early senescence and fungal disease resistance mechanisms.

An extensive study explored signatures of adaptations to the selection of the local environment based on the relationship between phenotypic adaptations (p-adaptations) and gene expression adaptations (e-adaptations) in the Arabidopsis population, utilizing extensive multiomic data from the 1001 Genomes Project [193]. Notably, enrichment analyses identified specific e-adaptations in Arabidopsis accessions from Central Asia and Southern Siberia, where genes such as *PATHOGENESIS-RELATED GENE 1* (*PR1*), *PLANT NATRIURETIC PEPTIDE* A (*PNP-A*), *APOPLASTIC* EDS1-*DEPENDENT 1* (*AED1*), and *RECEPTOR LIKE PROTEIN 23* (*RLP23*) were linked to enhanced systemic acquired resistance against oomycete and fungal detection. Correspondingly, a phenotype associated with leaf chlorosis was interpreted as a penetration-resistance response to microbial invasion, facilitating nutrient reallocation during the short growing season typical of these regions. This adaptive mechanism accelerates leaf senescence during reproduction, directing more nutrients to seed development, which is critical for survival in these climates [194].

Another comprehensive analysis of metaQTLs compiled from published literature revealed key genetic regions linked to various types of senescence and resistance to spot blotch, a critical disease in bread wheat caused by *B. sorokiniana* fungi [195]. A total of 16 consensus metaQTL regions were identified, consistently contributing to resistance across diverse genetic backgrounds and environments, including 12 multitrait metaQTLs. Notably, QTLs associated with delayed leaf senescence frequently overlapped with those linked to enhanced resistance to spot blotch, suggesting that slower senescence may confer resistance by prolonging photosynthetic activity. Within these QTL regions, several candidate genes involved in defense responses, signal transduction, and cell wall reinforcement were identified. These findings offer valuable genetic markers for breeding wheat varieties with improved resistance to spot blotch, potentially leading to better yield stability and resilience in disease-affected areas.

Overall, biotic stress-induced senescence in natural populations of model plants and crop varieties highlights the balance between defense mechanisms and growth, offering insights into genetic variation and survival strategies in diverse environments. In crop breeding, harnessing delayed senescence can improve disease resistance and yield stability, making crops more resilient to biotic stresses while maintaining productivity.

## 5. Challenges and Future Directions

### 5.1. Expanding Research Beyond Model Species: Understanding Leaf Senescence in a Broader Range of Plants

Most studies on the natural variations in leaf senescence have focused on model plants like Arabidopsis because of its rich genomic resources, short life cycle, efficient transformation system, and large research community. However, a significant gap remains in our understanding of leaf senescence across a broader range of species, particularly those of ecological and agronomical importance.

Future research should prioritize exploring crop wild relatives (CWRs), which are likely to harbor rich genetic variations that could shape senescence programs in response to environmental conditions. CWRs, the ancestors or progenitors of domesticated crop species and other close relatives throughout evolutionary history, contain genetic variants that may have been lost during domestication [196]. These wild species persist in their natural habitats, often located in and near the centers of origin [197], and are widespread across all continents except Antarctica. Several can be found in Vavilov’s center of origin and their adjacent regions [198]. Over millennia, these wild species have evolved under diverse environmental conditions, frequently developing traits that enhance resilience to various abiotic stresses. Thus, CWRs represent valuable reservoirs of genetic diversity that could contribute to improving adaptive traits linked to leaf senescence, such as the SG phenotypes, which are crucial under various stress conditions, including drought or heat stress. CWRs are taxonomically related to domesticated plants, often belonging to the same species or genus, and can naturally cross with cultivated species. This characteristic makes it possible to incorporate valuable genetic variants involved in senescence-related processes into domesticated crops [198,199,200,201]. For instance, hundreds of Ethiopian sorghum landraces in the drought-prone regions of Northeastern Africa have been explored to identify novel germplasm sources for drought tolerance based on their SG phenotypes [200]. Studies on wild potato germplasm have identified traits related to tuber starch content and N utilization efficiency, which could offer additional agronomic benefits [201].

Domestication and modern breeding practices have often reduced the genetic diversity of crop species, inadvertently eliminating traits that could confer greater fitness in natural environments, such as adaptive leaf senescence responses. As a result, many crops now possess limited genetic diversity and may lack the natural variation necessary for optimizing leaf senescence in response to changing environments. By exploring the extensive genetic diversity of CWRs and their diverse senescence responses, reintroducing these traits into cultivated species could help restore traits that confer greater resilience, such as stress-induced delayed senescence, ultimately enhancing agricultural sustainability under climate change.

### 5.2. Shifting from Single Stress to Multifactorial Stress Combinations to Evaluate Natural Senescence Programs

Most current studies that are aimed at identifying and evaluating the genetic basis of leaf senescence focus on individual senescence-inducing factors, which often fail to capture the complexity and variability of natural environments. However, plants typically face multiple stress factors simultaneously in nature, and these combined stresses significantly influence leaf senescence and overall plant fitness in more complex ways than single stressors [202,203,204].

The combination of shading and waterlogging or extreme temperature and drought in summer maize accelerates leaf senescence, disrupts photosynthetic activity, and reduces photoassimilate accumulation, decreasing yields [205,206]. Similarly, combined cold and drought stresses negatively influence leaf senescence and quality in tea plants [207], while combined drought and ozone stress negatively affect photosynthesis and accelerate senescence in wheat [208]. A recent study by Zandalinas et al. [209] investigated the impact of multifactorial stress combinations on plant growth and survival using Arabidopsis genotypes exposed to six different stresses—heat, salt, excess light, acidity, heavy metal, and oxidative stress—applied in various combinations under controlled conditions. Their findings highlighted the central role of ROS metabolism in plant resilience to combined stresses. Importantly, the transcriptomic response to stress combinations was unique and could not be accurately predicted based on the plant’s response to each stressor. High-order stress combinations, involving three or more simultaneous stresses, triggered the upregulation of a distinct set of genes not typically activated by individual stresses, while many well-established stress response pathways were suppressed.

This finding suggests that plants undergo fundamentally different genetic reprogramming when exposed to complex stress environments. This reprogramming underscores the complexity of plant adaptation to multifactorial stress and the importance of studying interactions between multiple stressors to better understand plant resilience [210]. Understanding these stress interactions is essential to uncovering the physiological and molecular mechanisms that regulate leaf senescence and identifying the genetic variants that contribute to multistress resilience. Although challenging, such studies provide valuable insights for developing crops with optimized senescence traits tailored to withstand the multifaceted challenges posed by climate change and other environmental pressures. Future research should focus on the specific and combined environmental challenges that individual plant species encounter in natural field conditions. By closely mimicking these natural stress conditions, this approach can accelerate the development of crops with improved resilience and adaptability to the diverse challenges posed by climate change.

### 5.3. From Technical Innovations to In-Depth Genetic Insights: Advancing Our Understanding of Senescence Regulation Through Omics Approaches and Gene Editing

Standardized populations enable researchers to employ high-throughput multiomic approaches across diverse environments using genomic information. These approaches, including epigenomics, transcriptomics, proteomics, metabolomics, and phenomics, offer a comprehensive understanding of the complex processes driving leaf senescence [211]. GWAS have gained widespread application for large population analysis, and mGWAS are increasingly being employed in this field.

In addition to these established approaches, numerous omics strategies can be incorporated. Epigenomic analysis revealed how modifications like DNA methylation and histone changes regulate gene activity without altering the DNA sequence. Mapping these modifications across different accessions allows researchers to link specific epigenetic signatures to senescence traits, providing critical data for GWAS to identify epigenetic QTLs associated with stress responses. However, transcriptomics uncovers gene expression profiles across accessions, highlighting the dynamic regulation of genes involved in senescence [212]. Integrating these data with GWAS allows for the identification of expression QTLs, which link genetic variants to the regulation of senescence-related genes.

Similarly, proteomics provides insights into the abundance and post-translational modifications of proteins involved in senescence regulation [213,214]. These data can be utilized to map protein QTLs, connecting genetic variants to changes in protein expression and activity during senescence. Finally, phenomics captures high-throughput physiological and morphological traits, such as photosynthetic efficiency and Chl content, which characterize the progression of senescence [66,67].

These multiomic approaches offer novel analytical dimensions to dissect the complex biological and molecular processes associated with senescence. Moreover, integrating multiomic data into association studies facilitates the exploration of the genetic foundations underlying the multiple processes involved in senescence, enhancing the comprehensive evaluation of senescence mechanisms.

In a different context, despite the identification of numerous candidate genes linked to genetic diversity in leaf senescence through GWAS and QTL fine mapping, substantial gaps remain in the functional validation of these genetic variants within natural populations or cultivars. Bridging this gap requires a shift from merely discovering candidate genes to elucidating the biological implications of these genetic variants in specific accessions or cultivars that possess them.

Functional validation is critical to confirm the roles of these genes and their natural allelic variants in regulating senescence processes [67,73]. The continuous advancement of CRISPR-based technologies, including precise double-strand breaks and cytosine and adenine base editing, offers powerful tools to generate loss-of-function mutations or nucleotide substitutions that mimic naturally occurring genetic variants [215]. These approaches enable the *in planta* evaluation of the biological function of specific variants in senescence regulation.

Additionally, investigating these genes across various genetic backgrounds and environmental conditions is critical for understanding how natural variation contributes to differential senescence responses in diverse plant populations. Molecular and genetic evaluation of senescence regulation in varying genetic backgrounds and environments will offer deeper insights into the adaptive significance of leaf senescence and its potential manipulation for crop improvement.

## 6. Conclusions

This review highlights the substantial contributions of natural genetic diversity to our understanding of leaf senescence, emphasizing its significance in fundamental plant biology and agricultural applications. By examining the genetic basis of diverse senescence programs across different species, we have illustrated the implication of complex genetic programs and environmental interactions that underlie the diversity of senescence programs (Table 1).

The natural variation in leaf senescence traits has provided a rich resource for identifying the key genes, alleles, and pathways involved in senescence regulation. However, several research gaps remain in fully translating these insights into practical breeding strategies. Current studies have primarily focused on model species and single stress conditions, which do not capture the full complexity of natural settings where multiple interacting stress factors influence leaf senescence. Additionally, the difficulty of transferring the findings from model species to a broader range of ecologically and agronomically important species presents significant barriers. Future research should prioritize expanding studies to include a wider variety of species, especially crop wild relatives, and identifying common genetic variants under multistress combinations. Moreover, integrative multiomic data with association analysis and advanced gene-editing technologies will be crucial to effectively harness natural genetic diversity to improve crop resilience and productivity.

Continued research and innovation in this field are essential to optimize leaf senescence in crops to balance yield, quality, and stress tolerance. As we face the challenges of climate change and global food security, leveraging the natural genetic diversity of plants to enhance crop resilience and productivity will be crucial for sustainable agriculture. By exploring new avenues in genetics, genome biology, and molecular breeding, we can unlock the full potential of natural variation to develop crops that are high-yielding, nutritious, and resilient to the changing global climate.

## Figures and Tables

**Table 1:** List of Genetic Variants Associated with Leaf Senescence Diversity.

Species	Gene (QTL)	Gene Description	Gene Effect ^1^	Germplasm ^3^	Factors ^6^	Ref. ^7^
		Biochemical Function	Allelic Effect (High/Low) ^2^	Approaches ^4,5^		
Arabidopsis	*ACD6*	Accelerated cell death 6	Positive	234 lines	Age, SA, Biotic stress	[67,187]
		Novel ankyrin transmembrane protein	SNP^8,297,892^ “G”-type/“T”-type	GWAS		
	*GVS1*	Genetic variants in leaf senescence 1	Positive	259 lines	Age, Dark	[24]
		Pinoresinol-lariciresinol reductase	SNP^16,500,847^ “C”-type/“A”-type	GWAS		
	*NMR19*	Naturally occurring DNA methylation variation region 19	Negative	137 lines	Dark	[116]
		Retrotransposon	NMR19-4m/NMR19-4u	WGBS		
	*TAT1*	Tyrosine aminotransferase 1	Negative	252 lines	Dark	[65]
		Cytosolic tyrosine aminotransferase	SNP^21,909,782^ “G”-type/“A”-type	mGWAS		
	*THA1*	Threonine aldolase 1	Negative	252 lines	Dark	[65]
		Threonine aldolase	SNP^2,725,344^ “A”-type/“G”-type	mGWAS		
	*NRT1.1*	Nitrate transporter 1.1	Negative	52 lines	N deficiency	[119]
		Dual-affinity nitrate transporter	7 bp del or A→C SNP^−1840^ /Col-0-type	GWAS		
	*CBF2* (*FTQ4*)	C-repeat binding factor 2	Negative	132 L*er*/Cvi RIL	Age, LT	[165,216]
		DREB subfamily A-1 of ERF/AP2 TF family	L*er*-type /Cvi-type (1630 bp del)	QTL		
Rice	*OsGW2* (*qCC2*)	Grain width and weight 2	Positive	*O. sativa*/ *O. grandiglumis* NIL	Age, Dark	[91]
		RING-type E3 ubiquitin ligase	*O. sativa*-type /*O. grandiglumis*-type	QTL, RNA-seq		
	*OsSGR*	Stay-green	Positive	141 indica /japonica RIL	Age	[93]
		Chlorophyll-degrading Mg^2+^-dechelatase	indica-type/japonica-type	QTL		
	*GHD7*	Grain number, plant height, and heading date 7	Positive	529 lines	Age	[99,217]
		CO, CO-LIKE, and TOC1 (CCT) domain proteins	(HAP-2, HAP-3, HAP-4) /HAP-1	GWAS		
	*NAL1* (*q* *LSCHL4*)	Narrow leaf 1	Positive	529 lines	Age	[99,103]
		Putative trypsin-like serine/cysteine protease	HAP-3/HAP-1	GWAS		
	*OsSG1*	Stay-green 1	Positive	368 lines	Age	[59]
		Glutamate-cysteine ligase	(HAP-2, HAP-3) /HAP-1	GWAS		
	*ARE1*	Abc1-1 repressor1	Positive	2155 lines	N deficiency	[125]
		Chloroplast protein	HAP-NPB /(HAP-9311, HAP-MH63)	Genetic mutant Seq analysis		
Wheat	*TaNAM-A1* (*qGLD-6A*)	No apical meristem-A1	Positive	480 178A/178B RIL	Age, N deficiency	[85]
		NAC transcription factor	HAP-A1a /HAP-A1d (1 bp del, C→T SNP^731^)	QTL		
	*ELS1*	Early leaf senescence 1	Negative	M114 × W301 RIL	Age, Biotic	[218]
		NLR protein	SNP^1308^ and SNP^1684^ “GC”-type/“TA”-type	Bulked segregant analysis, RNA-Seq		
Barley	*MLO*	Mildew locus O	Negative	NA	Age, Biotic	[191,192]
		Transmembrane protein	Allele *mlo-11*(*cnv2*) /*mlo-11*	Targeted seq		
Maize	*Zm00001* *d043586*	*AtS40-1* homologous	Positive	672 MAGIC lines	Age	[43,219]
		Protein of unknow function, DUF584	SNP^201,538,092^ “A”-type/“G”-type	GWAS		
Potato	*StCDF1*	Cycling DOF factor 1	Negative	762 lines	Age	[96,97,98]
		DNA-binding with one finger TF family	HAP-1/ (HAP-2, HAP-3)	GWAS		
Cucumber	*CsSGR*	Stay-green	Positive	173 lines	LT, Drought, Biotic stress	[173]
		Chlorophyll-degrading Mg^2+^-dechelatase	HAP-A/HAP-G	GWAS		
Cotton	*GhMKK9*	MAP kinase kinase 9	Positive	355 lines	Age, Drought	[60]
		Member of MAP kinase kinase family	HAP-2/HAP-1	GWAS		

^1^ Gene effect on the regulation of senescence is based on functional validation using overexpression or knockout approaches. ^2^ Allelic effect (High/Low) indicates high and low functional activity on genetic variation in each accession, respectively. HAP, Haplotype. ^3^ Number of germplasm lines, including accessions and artificial recombinant lines described in this review. ^4^ GWAS, genome-wide association studies; mGWAS, metabolite-based GWAS; WGBS, whole-genome bisulfite-sequencing; QTL, quantitative trait loci. ^5^ NA, not available. ^6^ LT, low temperature. ^7^ Ref., Reference.

## Data Availability

No new data were created or analyzed in this study.
